# Prolyl 4‐hydroxylase α‐subunit family regulation of type I collagen deposition and IL17RB/c‐Jun activation synergistically mediate choline dehydrogenase promotion of colorectal cancer metastasis

**DOI:** 10.1002/mco2.70007

**Published:** 2025-01-03

**Authors:** Xiaowen Yang, Yifei Li, Xinzhuang Shen, Shuying Wang, Zhuqing Zhang, Wenfei Du, Chenglong Yang, Xinyu Jiang, Xiaoyuan Zhang, Yongming Huang, Wenzhi Shen

**Affiliations:** ^1^ Cheeloo College of Medicine Shandong University Jinan China; ^2^ Shandong Provincial Precision Medicine Laboratory for Chronic Non‐communicable Diseases, Institute of Precision Medicine Jining Medical University Jining China; ^3^ Department of Oncology and Southwest Cancer Center Southwest Hospital, Third Military Medical University (Army Medical University) Chongqing China; ^4^ Department of Oncology The Seventh Medical Center of Chinese PLA General Hospital Beijing China; ^5^ Department of General Surgery Affiliated Hospital of Jining Medical University, Jining Medical University Jining China

**Keywords:** CHDH, collagen I, colorectal cancer (CRC), IL17RB/c‐Jun, metastasis, migration, P4HA

## Abstract

Metastasis continues to pose a significant challenge in tumor treatment. Evidence indicates that choline dehydrogenase (CHDH) is crucial in tumorigenesis. However, the functional role of CHDH in colorectal cancer (CRC) metastasis remains unreported. The study explored the functional role and mechanism of CHDH in CRC metastasis using human CRC tissues and a xenograft mouse model. CHDH expression was significantly higher in CRC compared to normal tissues and showed a positively correlation with CRC tumor‐nodes‐metastasis stage. CRC cell lines showed increased CHDH expression compared to normal controls. CHDH knockdown suppressed cell migration in vitro and tumor metastasis in vivo. Similarly, ectopic CHDH expression enhanced cell migration in vitro and tumor metastasis in vivo. Results suggested that CHDH affected the histone H3 trimethylation levels, which upregulated prolyl 4‐hydroxylase α‐subunit (P4HA) family gene (P4HA1/2/3) expression, further stabilizing collagen I expression and increasing IL17RB expression, which promoted downstream c‐Jun activation. Together, P4HA and IL17RB promote CRC cell metastasis. P4HA and c‐Jun inhibitors abolished CHDH‐mediated CRC cell metastasis in vitro and in vivo. Collectively, the above findings provide novel evidence that that CHDH mediates CRC cell metastasis and may be a promising target for metastatic CRC therapy.

## Graphical abstract

Choline dehydrogenase (CHDH), one of the enzymes involved in methyl donor pathway, affects histone H3 trimethylation, which in turn regulates the expression of the downstream target genes prolyl 4‐hydroxylase α‐subunits (P4HAs) and IL17RB. The P4HAs stabilize and promotes collagen I expression, and IL17RB promotes downstream c‐Jun activation, which together contribute to colorectal cancer (CRC) metastasis. 1,4‐Dihydrophenonthrolin‐4‐one‐3‐carboxylic acid (1,4‐DPCA) targets P4HAs, and SP600125 targets c‐Jun, both of which prevent CHDH‐mediated CRC metastasis

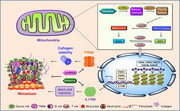

## 1 INTRODUCTION

Colorectal cancer (CRC) was historically an uncommon disease,^1^, ^2^ however, it is now one of the most prevalent malignant, which has the third and fourth highest incidence and mortality rates, respectively. Approximately 1.2 million patients worldwide are diagnosed and more than 900,000 patients die directly or indirectly from CRC each year.^3^ Metastasis, a key characteristic of cancer, is the leading cause of CRC‐related deaths and poses a significant challenge in clinical CRC treatment.^4^, ^5^ Therefore, uncovering the molecular markers related to tumor metastasis and establishing the biological mechanisms of the metastatic process are crucial for identifying therapeutic windows for successful intervention.

Human choline dehydrogenase (CHDH) is a mitochondrial enzyme that is involved in choline metabolism.^6^, ^7^ The CHDH gene is situated at 3p21.1 on chromosome 3.^8^ In mitochondria, CHDH regulates blood and cellular concentrations of choline and glycine by catalyzing the oxidation of choline to glycine betaine. Choline plays an integral role in several biological processes in the body, such as the biosynthesis of acetylcholine. Glycine betaine, on the other hand, maintains methyl donor acceptor homeostasis for the S‐adenosylmethionine to S‐adenosylhomocysteine process by affecting the amount of homocysteine.^6^ Recent studies have revealed that CHDH is associated with the development of various human pathologies, including male infertility,^9^, ^10^ homocystinuria,^11^ breast cancer,^12^, ^13^ and metabolic syndrome,^14^, ^15^ and that CHDH plays a crucial role in mitochondrial autophagy.^16^ CHDH is a candidate oncogene linked to the development of breast, pancreatic,^17^ head and neck squamous cell carcinoma,^18^ and renal cell carcinoma.^19^, ^20^ There is also evidence that CHDH may be associated with CRC metastasis or epithelial–mesenchymal transition (EMT),^21^ nevertheless, the specific functional and molecular mechanisms of CHDH in CRC metastasis remain unknown.

Prolyl hydroxylation, a prevalent post‐translational modification, regulates protein folding and stability in mammalian cells. A large proportion of hydroxyproline is found in collagen residues in animal proteins.^22^ Prolyl 4‐hydroxylase (P4H) is a 22‐tetrameric ketoglutarate‐dependent dioxygenase that catalyzes proline 4‐hydroxylation and facilitates collagen triple helix formation.^23^ It was found that P4H α‐subunit (P4HA) is responsible for both peptide binding and catalysis. Mammalian cells contain three P4HA isoforms (P4HA1‐3),^24^ and are major contributors to the P4H activity in cells and tissues.^25^ It has been demonstrated that an increase in collagen production is associated with the development and progression of various tumors; for example, P4HA expression increases significantly during breast carcinogenesis and progression, indicating a poor prognosis.^26^, ^27^ Interestingly, triple‐negative breast cancer (TNBC) cells that express P4HA1 are more likely to metastasize, stabilizes hypoxia inducible factor (HIF)‐1α expression, and maintains chemoresistance.^26^ A previous study has reported that P4HA2 enhances breast cancer progression and metastasis through collagen deposition regulation.^27^ In addition, it is reported that P4HA3 promotes clear cell renal cell carcinoma (ccRCC) progression via PI3K/AKT/GSK3β signaling.^28^ However, the role of P4HA in CHDH‐mediated CRC progression and metastasis has not yet been reported.

c‐Jun is the most active transcription factor in the activator protein‐1 complex.^29^ It regulates tumor cell growth in response to extracellular stimuli like growth factors, cytokines, and stress. Research indicates a strong correlation between elevated c‐Jun expression and the development and prognosis of several malignant tumors.^30^ As a key member of the mitogen‐activated protein kinases, activated c‐Jun N‐terminal kinase (JNK) recognizes and binds to the amino‐terminal region of c‐Jun, and bisphosphorylates serine‐63 and serine‐73 sites in the activation region of c‐Jun, which activates and enhances its transcriptional activity, thereby regulating tumor proliferation, apoptosis, and metastasis.^31^ As a classical inhibitor of JNK, SP600125 also inhibits c‐Jun activation,^32^ and has been used in antitumor therapy. Although the role of JNK/c‐Jun in tumors has been extensively studied, its role in CHDH‐mediated tumor progression remains unclear.

In the present study, we aimed to investigate the functional role and mechanism of CHDH in CRC metastasis. We found that CHDH is highly expressed in CRC and promotes cell migration in vitro and tumor metastasis in vivo. We revealed that CHDH affects histone H3 trimethylation, which upregulates the expression of P4HA family genes (P4HA1/2/3) to stabilize and promote the expression of collagen I and increases the expression of IL17RB to promote the downstream activation of c‐Jun, which together contribute to CRC metastasis. As a therapeutic strategy, we demonstrated that P4HA and c‐Jun inhibitors inhibited CHDH‐mediated CRC metastasis in vitro and in vivo. Our study identified novel markers of CRC metastasis and identified new targets and strategies for the treatment of CRC metastasis.

## 2 RESULTS

### 2.1 CHDH was upregulated in human CRC tissues

**FIGURE 1:**
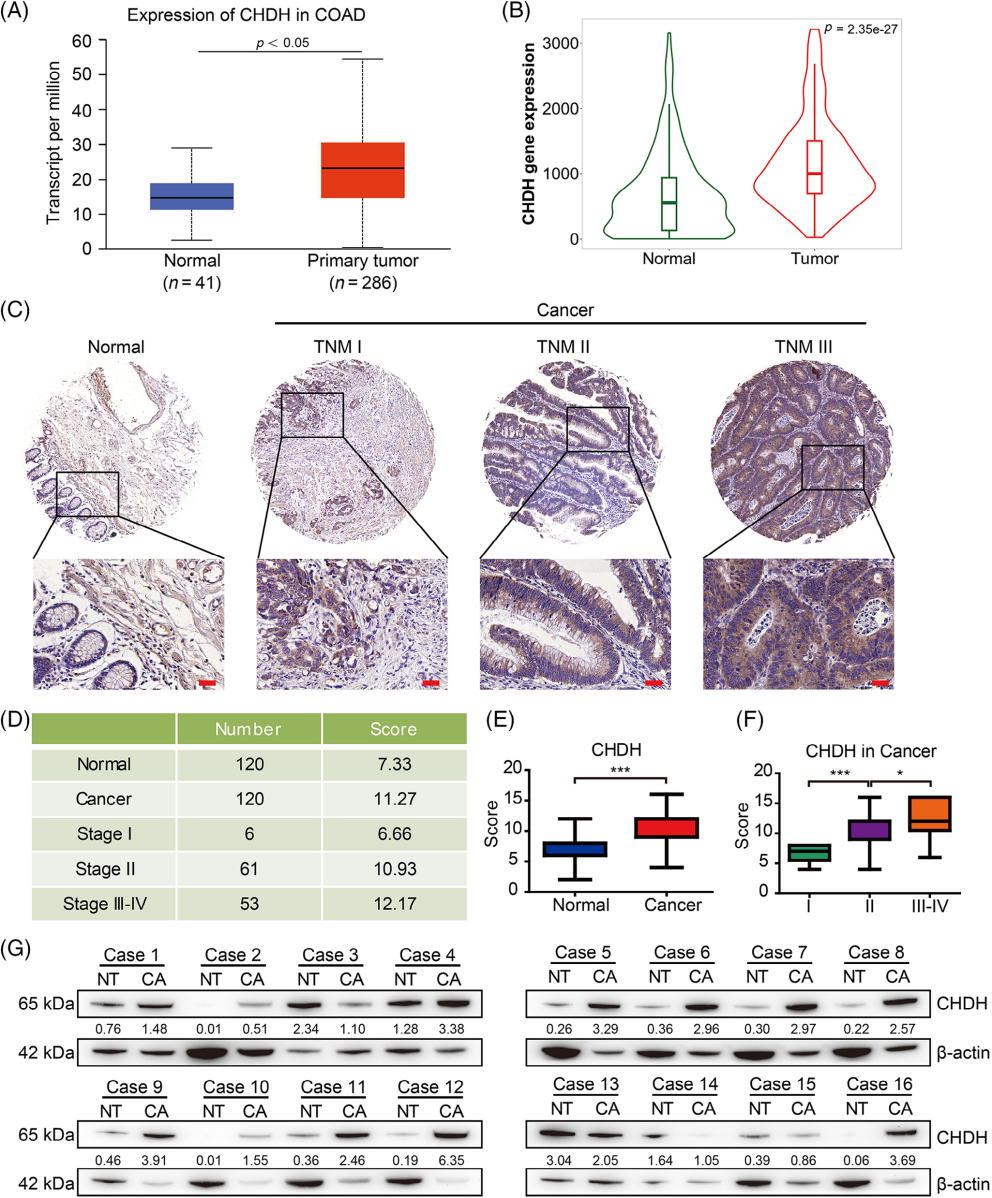
Choline dehydrogenase (CHDH) was highly expressed in human colorectal cancer (CRC) tissues. (A, B) UALCAN and TNMplot online public databases were used to analyze the transcriptional expression of CHDH in colon cancers (cancer vs. normal tissue, *p* < 0.05). (C) Representative immunohistochemistry (IHC) images of CHDH expression in tissues of different states and stages in human CRC tissue microarray. Scale bar: 50 µm. (D) Quantitative results of CHDH expression in tissue array of different states and stages were shown. (E, F) The correlation of CHDH expression and tumor‐nodes‐metastases (TNMs) was quantified. *p < 0.05, ***p < 0.001. (G) Expression of CHDH in 16 pairs of clinical patient‐derived colon cancer tissues and adjacent normal tissues was analyzed by western blotting. CA, cancer tissue; COAD, colon adenocarcinoma; NT, normal tissue.

To explore the expression pattern of CHDH in human CRC, we first performed bioinformatics analysis using the UALCAN and TNMplot online public databases. The results indicated that the expression of CHDH in CRC tissues was significantly higher than that in normal tissues in these two public databases (Figure 1A,B, *p* < 0.05). To validate these findings, we performed immunohistochemistry (IHC) analysis of human CRC tissue microarrays containing 240 samples (120 normal colon tissues and 120 CRC tissues) using CHDH‐specific antibodies. The results showed that CHDH expression was elevated in tumor biopsy tissues compared to that in normal tissues (Figure 1C–E). We also investigated the correlation between CHDH expression and tumor‐nodes‐metastasis (TNM) stage of tumor biopsies. We noticed that elevated CHDH expression levels positively correlated with tumor biopsies with a high TNM stage (Figure 1F). Moreover, we examined the expression of CHDH in 16 pairs of CRC tissues and adjacent nontumor tissues by western blotting. The blotting results suggested that CHDH was highly expressed in CRC tissues and rarely expressed in adjacent nontumor tissues (Figure 1G). Taken together, these findings suggest that CHDH is upregulated as a potential oncogene in human CRC tissues.

### 2.2 CHDH deficiency inhibited tumor cell migration in vitro

**FIGURE 2:**
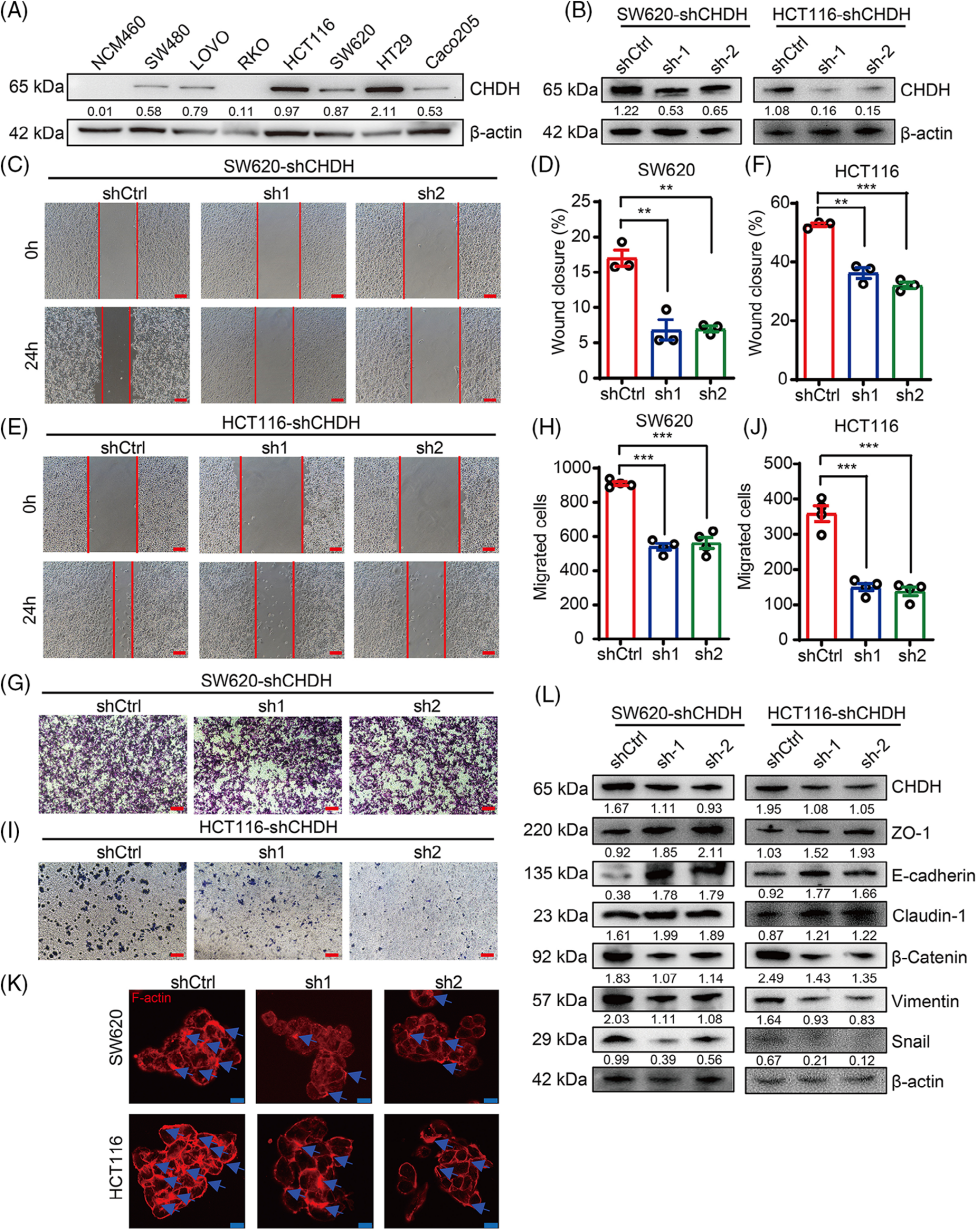
Choline dehydrogenase (CHDH) silencing suppresses colorectal cancer (CRC) cell migration in vitro. (A) Western blot analysis of CHDH expression in normal colonic epithelial cell line and CRC cell lines. (B) Western blot analysis CHDH knockdown efficiency in SW620 and HCT116 cell lines. (C) Wound‐healing assay images of SW620‐shCHDH or shctrl were shown. Scale bar: 50 µm. (D) Statistic results on wound closure of SW620‐shCHDH or shctrl. **p < 0.01. (E) Wound‐healing assay images of HCT116‐shCHDH or shctrl were shown. Scale bar: 50 µm. (F) Statistic results on wound closure of HCT116‐shCHDH or shctrl. **p < 0.01, ***p < 0.001. (G) Transwell assay images of SW620‐shCHDH or shctrl were shown. Scale bar: 50 µm. (H) Statistic results on migrated cells of SW620‐shCHDH or shctrl. ***p < 0.001. (I) Transwell assay images of HCT116‐shCHDH or shctrl were shown. Scale bar: 50 µm. (J) Statistic results on migrated cells of HCT116‐shCHDH or shctrl. ***p < 0.001. (K) Immunofluorescence assay to detect F‐actin rearrangement in SW620 and HCT116 expressing shCHDH or shctrl cells via phalloidin staining. Scale bar: 20 µm. (L) Western blot analysis the expression of epithelial–mesenchymal transition (EMT) markers in SW620 and HCT116 expressing shCHDH or shctrl cells.

Further we analyzed the expression of CHDH in colon epithelial cells and CRC cell lines. Consistent with the expression in CRC tissues, CHDH protein levels were upregulated in CRC cell lines compared to those in normal colon cells, particularly in HCT116 and SW620 cells (Figure 2A). Thus, these cell lines appear to be suitable models to determine the effect of CHDH on tumor migration in vitro.

To explore the functional role of CHDH in tumor migration in vitro, we knocked down CHDH expression in HCT116 and SW620 cells using CHDH‐specific short hairpin RNAs (shRNAs; Figure 2B). The wound‐healing assay results displayed that the CHDH knockdown suppressed the migration ability of HCT116 and SW620 cells (Figure 2C–F). Transwell assays demonstrated that CHDH knockdown reduced the migration ability of both cell lines (Figure 2G–J). Immunofluorescence staining showed that CHDH knockdown decreased lamellipodia formation and cluster formation (Figure 2K). Moreover, the expression of metastasis‐associated EMT markers was detected. As shown in Figure 2L, CHDH knockdown reduced the expression of mesenchymal markers (vimentin, snail, and β‐catenin) and elevated the expression of epithelial markers (E‐cadherin, ZO‐1, and claudin‐1). Altogether, these findings suggest that CHDH acts a crucial role in regulating tumor migration in vitro.

### 2.3 Ectopic expression of CHDH promoted tumor migration in vitro

**FIGURE 3:**
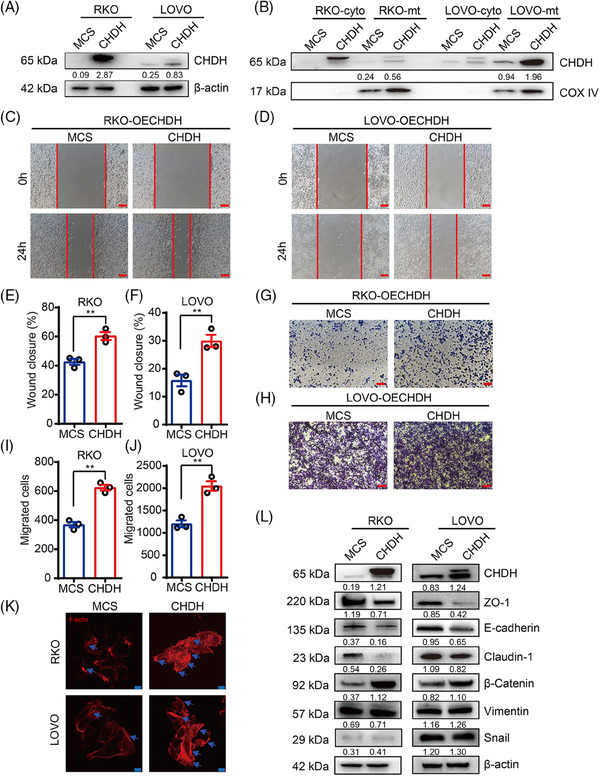
Ectopic expression of choline dehydrogenase (CHDH) facilitates colorectal cancer (CRC) cell migration in vitro. (A) Western blot analysis CHDH overexpression efficiency in RKO and LOVO cell lines. (B) Western blot analysis CHDH overexpression efficiency in cytoplasm (cyto) and mitochondria (mt). (C) Wound‐healing assay images of RKO expressing CHDH or multiple cloning site (MCS) were shown. Scale bar: 50 µm. (D) Wound‐healing assay images of LOVO expressing CHDH or MCS were shown. Scale bar: 50 µm. (E) Statistic results on wound closure of RKO expressing CHDH or MCS. **p < 0.01. (F) Statistic results on wound closure of LOVO expressing CHDH or MCS. **p < 0.01. (G) Transwell assay images of RKO expressing CHDH or MCS were shown. Scale bar: 50 µm. (H) Transwell assay images of LOVO expressing CHDH or MCS were shown. Scale bar: 50 µm. (I) Statistic results on migrated cells in RKO expressing CHDH or MCS. **p < 0.01. (J) Statistic results on migrated cells in LOVO expressing CHDH or MCS. **p < 0.01. (K) Immunofluorescence assay to detect F‐actin rearrangement in RKO and LOVO expressing CHDH or MCS cells via phalloidin staining. Scale bar: 20 µm. (L) Western blot to analysis the expression of epithelial–mesenchymal transition (EMT) markers in RKO and LOVO expressing CHDH or MCS cells.

To further evaluate the effect of CHDH on tumor migration, we ectopically expressed CHDH in RKO and LOVO cells with low CHDH expression and found that the efficiency of CHDH overexpression was high, as detected using immunoblotting (Figure 3A). Based on the localization of CHDH, we extracted mitochondrial proteins and found that CHDH was also overexpressed in the mitochondria of CHDH overexpression cells compared with expression levels in normal cells (Figure 3B). The wound‐healing experiments demonstrated that ectopic CHDH expression enhanced cell migration in both RKO (Figure 3C,E) and LOVO (Figure 3D,F) cell lines compared with that of normal cells. The transwell assay obtained the same results, which ectopic CHDH expression enhanced the migration ability of RKO (Figure 3G,I) and LOVO (Figure 3H,J) cells. Moreover, immunofluorescence staining showed that ectopic expression of CHDH increased lamellipodia formation or cluster formation (Figure 3K). In addition, western blotting for EMT markers displayed that CHDH ectopic expression elevated the expression of vimentin, snail, and β‐catenin, and reduced the expression of E‐cadherin, ZO‐1, and claudin‐1 (Figure 3L). To verify the correlation between CHDH and the EMT markers (ZO‐1, E‐cadherin, N‐cadherin, and β‐catenin), IHC staining was conducted on 20 human CRC samples to assess their coexpression. The results revealed a positive correlation between CHDH and N‐cadherin/β‐catenin, a negative correlation between CHDH and E‐cadherin/ZO‐1 (Figure S1A). Collectively, the above results confirm the key effect of CHDH in promoting tumor migration.

### 2.4 CHDH promoted tumor metastasis in vivo

**FIGURE 4:**
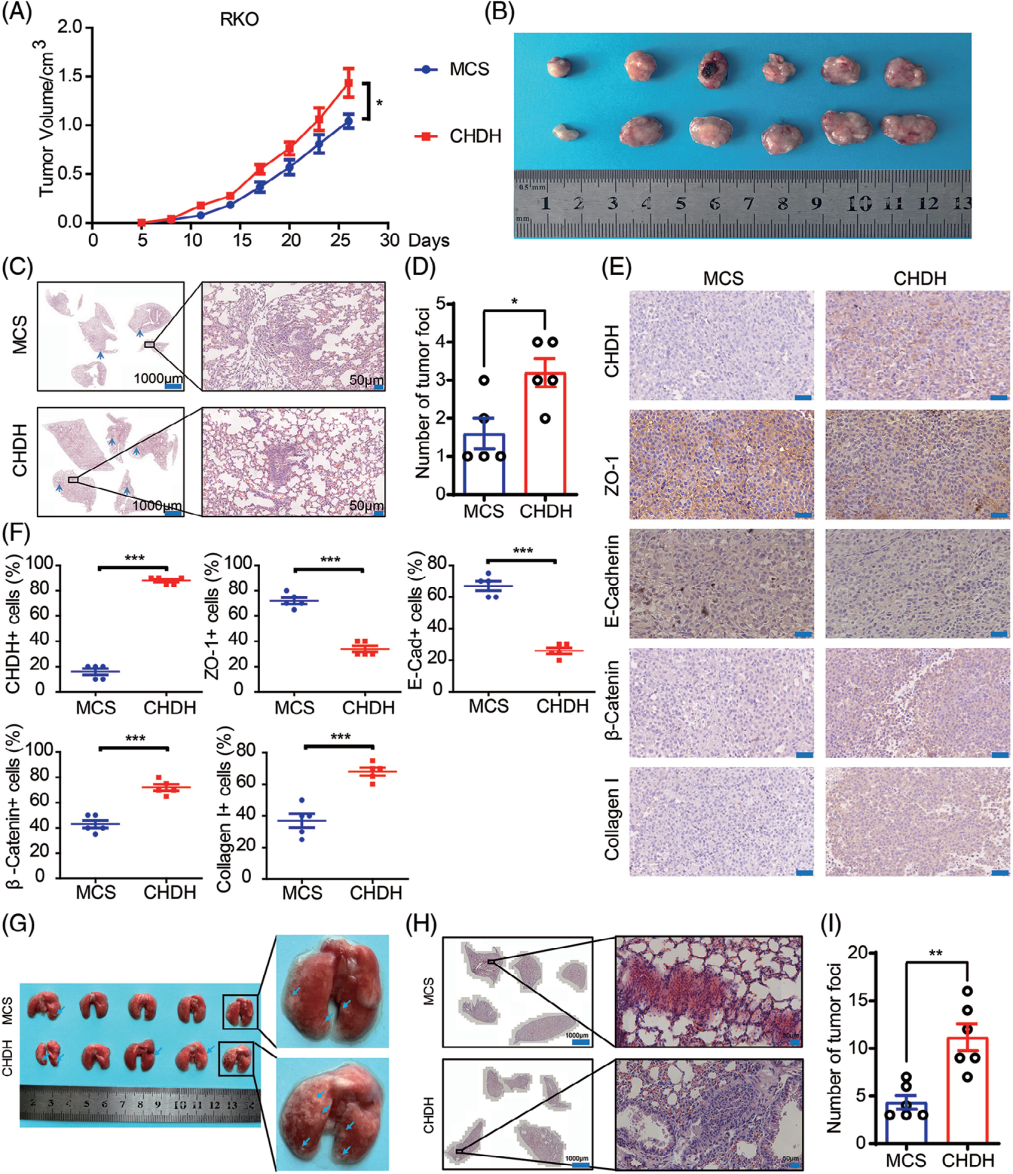
Ectopic expression of choline dehydrogenase (CHDH) promotes colorectal cancer (CRC) metastasis in vivo. (A) Stable RKO‐MCS and RKO‐CHDH cells were injected subcutaneously at the fourth fat pad in 5‐week‐old nude mice and tumor growth curves were recorded. *p < 0.05. (B) The tumors isolated from RKO‐MCS and RKO‐CHDH group mice were shown. (C) Representative hematoxylin and eosin (H&E) staining images of lungs from RKO‐MCS and RKO‐CHDH groups were shown. (D) A statistical analysis of lung metastatic nodules observed in RKO‐MCS and RKO‐CHDH groups was presented. *p < 0.05. (E) Immunohistochemistry (IHC) staining assay was conducted to check the expression of CHDH, ZO‐1, E‐cadherin, β‐catenin, and collagen I in various group mice tumors. Scale bar: 50 µm. (F) The statistical results of CHDH, ZO‐1, E‐cadherin, β‐catenin, and collagen I positive cells in various group mice tumors were shown. ***p < 0.001. (G) Construction of a mouse tail vein‐lung metastasis model and representative images of lungs from RKO‐MCS and RKO‐CHDH groups were shown. (H) Representative H&E staining images of lungs from RKO‐MCS and RKO‐CHDH groups were shown. (I) A statistical analysis of lung metastatic nodules observed in RKO‐MCS and RKO‐CHDH groups was presented. **p < 0.01.

Given that CHDH has the property of promoting tumor cell metastasis in vitro, we investigated the corresponding in vivo effects in a xenograft mouse model. Stable RKO‐multiple cloning site (MCS) and RKO‐CHDH cells were injected into the anterior fat pads of nude mice to generate xenografts. As shown in Figure 4A, tumor growth was significantly accelerated and tumor volume was significantly increased in the RKO‐CHDH group compared to the RKO‐MCS group (Figure 4B). Additionally, RKO‐CHDH mice showed more lung metastatic nodules and areas compared with RKO‐MCS mice in hematoxylin and eosin (H&E) staining of the lungs (Figure 4C,D). IHC results showed that epithelial marker expression was significantly decreased and mesenchymal marker expression was significantly increased in the tumors of the RKO‐CHDH group (Figure 4E,F). To further confirm the above results, RKO‐MCS and RKO‐CHDH cells were separately injected into the tail vein to construct a lung metastasis model. As shown in Figure 4G,H, compared with the RKO‐MCS group, the RKO‐CHDH group showed significantly more lung metastatic nodules, and the area of metastatic nodules was significantly larger. Collectively, our results suggest that CHDH promotes tumor metastasis in vivo.

### 2.5 CHDH promoted the expression of P4HA by altering the methylation of histone H3, which ultimately mediated CRC cell migration

**FIGURE 5:**
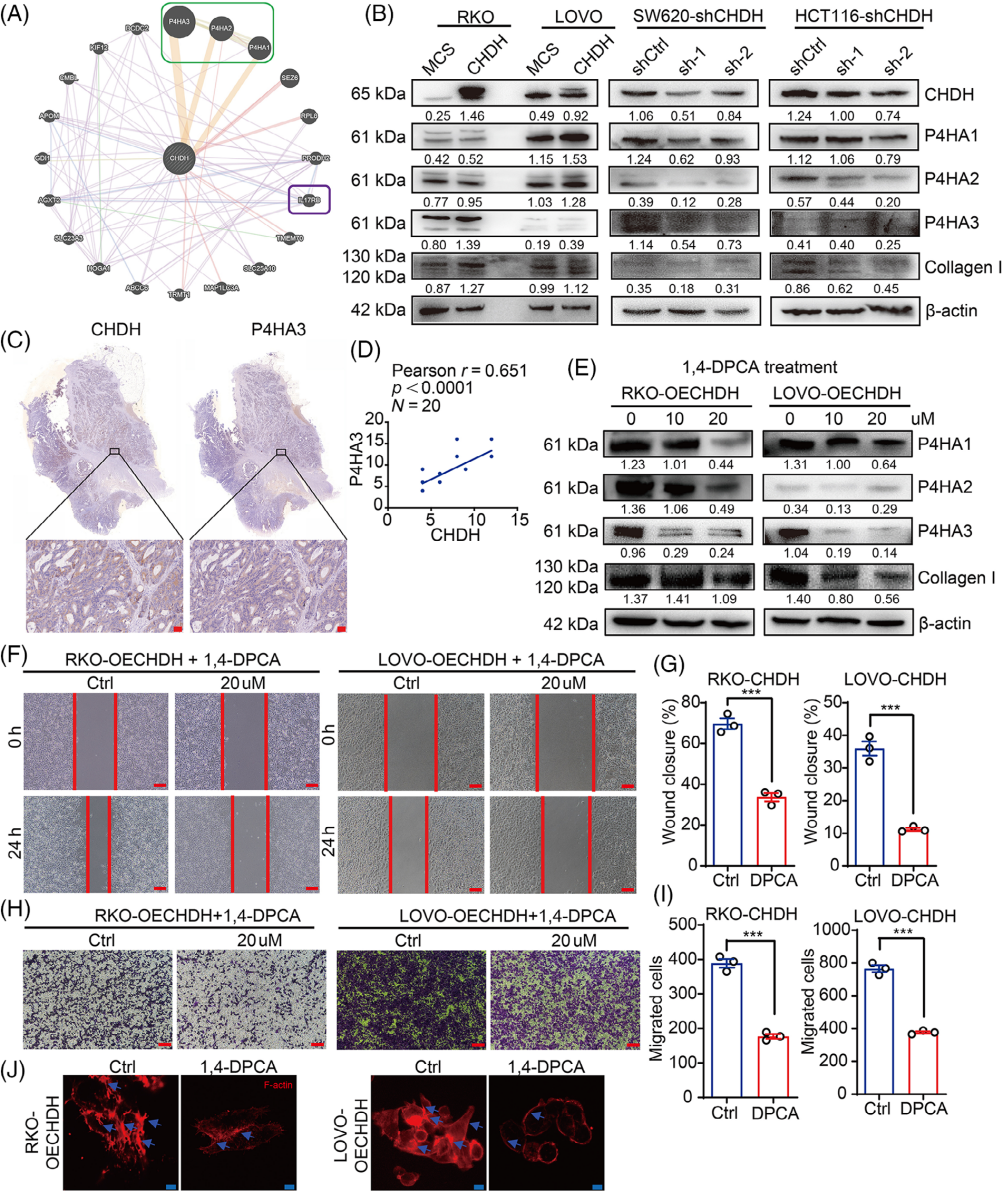
Choline dehydrogenase (CHDH) promotes the prolyl 4‐hydroxylase α‐subunits (P4HAs) expression by altering the methylation of histone H3, which ultimately mediates colorectal cancer (CRC) cell migration. (A) GENEMANIA online database were used to analyze CHDH coexpression genes. (B) Western blot analysis the expression of P4HA1/2/3 and collagen I in CHDH knockdown or overexpression cells. (C, D) Representative immunohistochemistry (IHC) staining of CHDH and P4HA3 in human CRC tissues and the correlation results were shown, scale bar: 50 µm. (E) Western blot analysis the expression of P4HA1/2/3 and collagen I in CHDH overexpression cells treated with 1,4‐dihydrophenonthrolin‐4‐one‐3‐carboxylic acid (1,4‐DPCA). (F) Wound‐healing assay images of RKO and LOVO expressing CHDH cells treated with DMSO (Ctrl) or 1,4‐DPCA were shown. Scale bar: 50 µm. (G) Statistic results on wound closure of RKO and LOVO expressing CHDH cells treated with DMSO (Ctrl) or 1,4‐DPCA. ***p < 0.001. (H) Transwell assay images of RKO and LOVO expressing CHDH cells treated with DMSO (Ctrl) or 1,4‐DPCA were shown. Scale bar: 50 µm. (I) Statistic results on migrated cells in RKO and LOVO expressing CHDH cells treated with DMSO (Ctrl) or 1,4‐DPCA. ***p < 0.001. (J) Immunofluorescence assay to detect F‐actin rearrangement in RKO and LOVO expressing CHDH cells treated with DMSO (Ctrl) or 1,4‐DPCA via phalloidin staining. Scale bar: 20 µm.

To determine the molecular mechanism underlying CHDH, we analyzed gene sets related to CHDH function using the GENEMANIA online database. Of the 20 genes related to CHDH function analyzed (Figure 5A), the most significant relationship observed was with the P4HA family genes. It has been previously demonstrated that the P4HA isoforms can prolyl hydroxylate collagen I, which in turn stabilizes collagen structure to promote tumor metastasis. Therefore, we focused on the function of the P4HAs in CHDH‐mediated metastasis of CRC. Compared to normal cells, western blotting of P4HA1‐3 and collagen I in CHDH knockdown and overexpression cell lines showed that P4HA1‐3 and collagen I expression was reduced with CHDH knockdown and increased with ectopic expression of CHDH (Figure 5B). To confirm the correlation between CHDH and the P4HAs, IHC staining was performed on 20 human CRC samples to detect the coexpression of CHDH and P4HA1/2/3. The results revealed a positive correlation between CHDH and P4HA3 (Figure 5C,D), P4HA2 (Figure S1B), and P4HA1 (Figure S1C). Moreover, TNMplot databases analysis results showed that P4HA1, P4HA2, and P4HA3 were highly expressed in CRC (Figure S1D–F).

To verify the effect of the P4HAs in CHDH‐mediated CRC cell migration, the P4HA‐specific small‐molecule inhibitor 1,4‐dihydrophenonthrolin‐4‐one‐3‐carboxylic acid (1,4‐DPCA) was used to inhibit P4HA expression. Western blotting results showed that 20 µM 1,4‐DPCA effectively inhibited the expression of P4HA and collagen I in RKO‐ and LOVO‐CHDH cells compared to cells without inhibitors (Figure 5E). The wound‐healing assay results showed that P4HA1/2/3 inhibition abolished CHDH‐mediated CRC cell migration (Figure 5F,G). The transwell assay results indicated that CHDH‐mediated cell migration was attenuated upon P4HA1/2/3 inhibition (Figure 5H,I). In addition, immunofluorescence staining results showed that CHDH‐mediated increased lamellipodia formation or cluster formation was reduced upon P4HA1/2/3 inhibition (Figure 5J).

Next, we focused on how CHDH affects the expression of the P4HAs. We first examined the interaction between CHDH and P4HA1/2/3 proteins by immunoprecipitation, which showed that CHDH did not interact with P4HA1/2/3 (Figure S2A). It has been previously shown that choline metabolism acts as one of the main methyl donor pathway and affects the methylation process.^7^, ^33^ Trimethylation of histone H3, a common form of methylation, regulates the expression of downstream genes. Therefore, we examined the effect of changes in CHDH expression on histone H3 trimethylation using western blotting and a methylation antibody kit. The results revealed that ectopic expression of CHDH affected the trimethylation of histone H3 at multiple sites (K4, K9, K27, K36, and K79) compared with that in normal cells (Figure S2B), which may in turn regulate the expression of downstream target genes, such as P4HA1/2/3.

In summary, these results reveal that CHDH promotes the expression of the P4HAs by altering the methylation of histone H3, which ultimately mediates CRC cell migration.

### 2.6 CHDH‐mediated IL17RB/c‐Jun signaling to promote CRC cell migration in vitro

**FIGURE 6:**
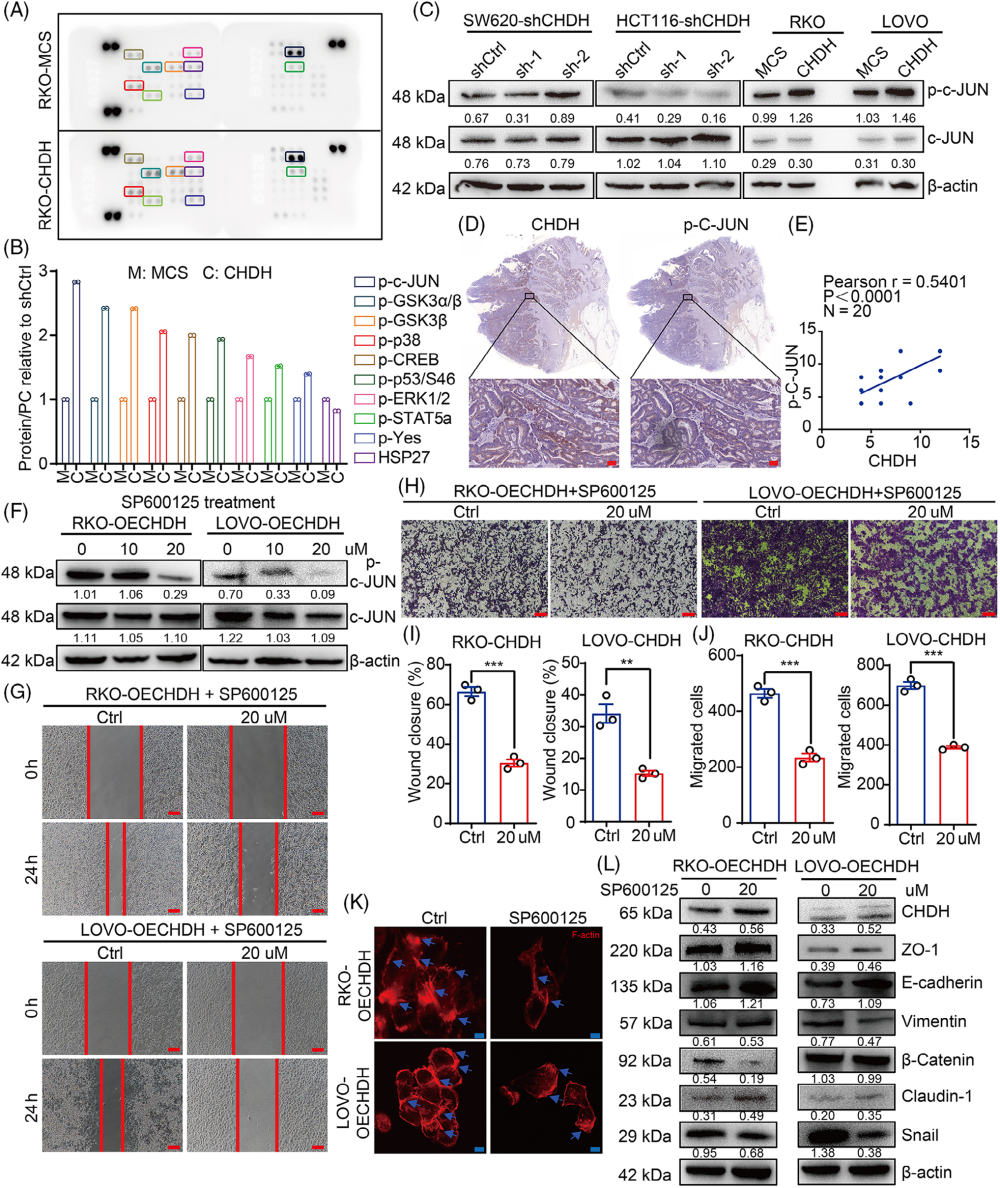
Choline dehydrogenase (CHDH) mediates c‐Jun activation to promote colorectal cancer (CRC) cell migration in vitro. (A, B) Activation of 39 kinases or key proteins were detected in RKO cells overexpressing CHDH or MCS using a human phosphokinase array. (C) Western blot analysis the expression of c‐Jun and p‐c‐Jun in CHDH knockdown or overexpression cells. (D, E) Representative immunohistochemistry (IHC) staining of CHDH and p‐c‐Jun in human CRC tissues and the correlation results were shown, scale bar: 50 µm. (F) Western blot analysis the expression of c‐Jun and p‐c‐Jun in CHDH overexpression cells treated with SP600125. (G) Wound‐healing assay images of RKO and LOVO expressing CHDH cells treated with DMSO (Ctrl) or SP600125 were shown. Scale bar: 50 µm. (H) Transwell assay images of RKO and LOVO expressing CHDH cells treated with DMSO (Ctrl) or SP600125 were shown. Scale bar: 50 µm. (I) Statistic results on wound closure in RKO and LOVO expressing CHDH cells treated with DMSO (Ctrl) or SP600125. **p < 0.01, ***p < 0.001. (J) Statistic results on migrated cells in RKO and LOVO expressing CHDH cells treated with DMSO (Ctrl) or SP600125. ***p < 0.001. (K) Immunofluorescence assay to detect F‐actin rearrangement in RKO and LOVO expressing CHDH cells treated with DMSO (Ctrl) or SP600125 via phalloidin staining. Scale bar: 20 µm. (L) Western blot analysis the expression of epithelial–mesenchymal transition (EMT) markers in RKO and LOVO expressing CHDH cells treated with DMSO (Ctrl) or SP600125.

To further determine the underlying mechanism by which CHDH mediates cell migration, we conducted human phosphokinase array analysis in RKO‐MCS and RKO‐CHDH cells, which contained 39 kinase phosphorylation patterns or key proteins. As shown in Figure 6A,B, ectopic expression of CHDH increased the expression p‐c‐Jun, compared with RKO‐MCS cells. Further we detected the activation of c‐Jun in CHDH knockdown and CHDH overexpression cell lines using immunoblotting. Compared with normal CRC cells, p‐c‐Jun expression was decreased after CHDH knockdown and increased with ectopic expression of CHDH (Figure 6C). These findings indicate that ectopic expression of CHDH promotes c‐Jun activation in vitro. To verify the correlation between CHDH and p‐c‐Jun, IHC staining was performed on 20 human CRC samples to evaluate coexpression. A positive correlation was observed between CHDH and p‐c‐Jun levels (Figure 6D,E).

To evaluate the effect of c‐Jun activation in CHDH‐mediated cell migration, the c‐Jun‐specific small‐molecule inhibitor SP600125 was used to suppress the phosphorylation of c‐Jun. Western blotting results displayed that 20 µM SP600125 effectively inhibited the activation of c‐Jun in RKO‐CHDH and LOVO‐CHDH cells (Figure 6F). The wound healing (Figure 6G,H) and transwell (Figure 6I,J) assays showed that p‐c‐Jun inhibition abolished CHDH‐mediated CRC cell migration. The immunofluorescence staining assay showed that CHDH‐mediated increased lamellipodia or cluster formation was reduced upon p‐c‐Jun inhibition (Figure 6K). In addition, western blotting results showed that the CHDH‐mediated changes in expression of EMT markers were markedly rescued by SP600125 treatment (Figure 6L).

To confirm the mechanism of c‐Jun activation by CHDH, we reanalyzed the CHDH‐related genes (Figure 5A) and found that *IL17RB*, a CHDH‐related gene, is also a classical upstream signaling molecule for c‐Jun activation. Based on this, we analyzed the expression of IL17RB in CRC using the TNMplot and UALCAN databases. IL17RB shows higher expression in CRC, which is consistent with CHDH (Figure S1G,H). We then detected the expression of *IL17RB* in CRC tissues with high CHDH expression using western blotting. The results demonstrated that IL17RB was upregulated in CHDH high expressed CRC tissues (Figure S3A), and that there was a positive correlation between CHDH and IL17RB (Figure S3B). TIMER database analyses yielded the same results (Figure S3C). Moreover, we tested the expression of IL17RB in CHDH deficient or overexpression cells. The results indicated that IL17RB expression changes with CHDH deficiency or overexpression (Figure S3D). We also examined the interaction between CHDH and c‐JUN or IL17RB proteins by immunoprecipitation (IP) and showed that CHDH did not interact with c‐JUN or IL17RB (Figure S2A).

To evaluate the role of IL17RB expression in c‐Jun activation and CHDH‐mediated CRC cell migration, the IL17RB‐specific antibody was used to block IL17RB expression. Western blotting results showed that IL17RB antibody effectively inhibited the activation of c‐Jun in RKO‐CHDH and LOVO‐CHDH cells (Figure S3E). The wound healing (Figure S3F,G) and transwell (Figure S3H,I) assays showed that IL17RB antibody blocking abolished CHDH‐mediated CRC cell migration. In addition, CHDH‐mediated expression of EMT markers was markedly rescued by IL17RB antibody blocking as well (Figure S3E).

Collectively, these results suggest that CHDH mediates IL17RB/c‐Jun signaling to promote CRC cell migration, and that c‐Jun activation is indispensable for CHDH‐mediated CRC cell migration in vitro.

### 2.7 Combined 1,4‐DPCA and SP600125 treatment eliminated CHDH‐mediated CRC cell migration in vitro

**FIGURE 7:**
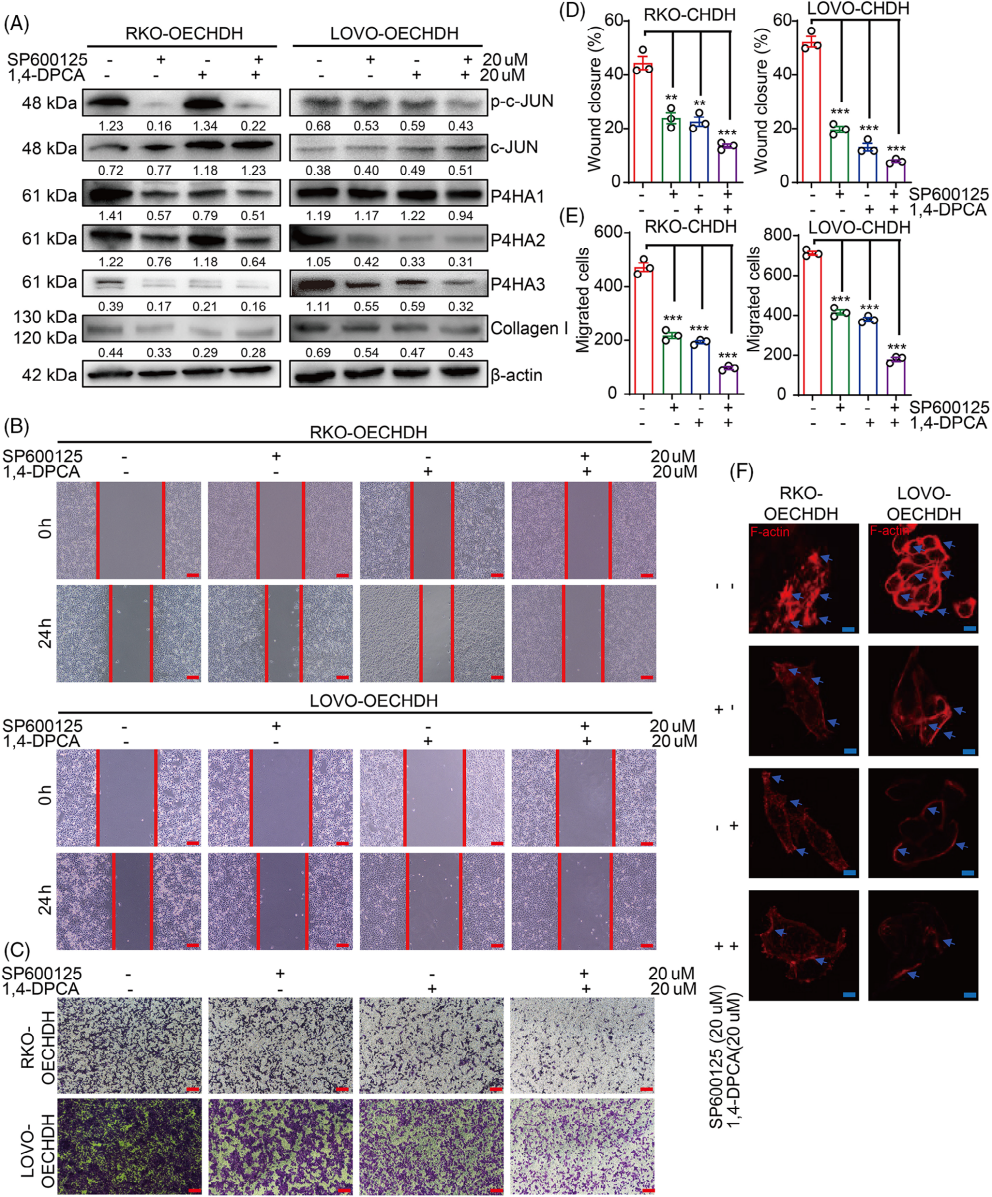
1,4‐Dihydrophenonthrolin‐4‐one‐3‐carboxylic acid (1,4‐DPCA) combined with SP600125 eliminates choline dehydrogenase (CHDH)‐mediated colorectal cancer (CRC) cell migration more effectively in vitro. (A) Western blot analysis the expression of c‐Jun, p‐c‐Jun, prolyl 4‐hydroxylase α‐subunits (P4HAs), and collagen I in RKO and LOVO expressing CHDH cells treated with 1,4‐DPCA or SP600125 or 1,4‐DPCA combine SP600125. (B) Wound‐healing assay images of RKO and LOVO expressing CHDH cells treated with 1,4‐DPCA or SP600125 or 1,4‐DPCA combine SP600125 were shown. Scale bar: 50 µm. (C) Transwell assay images of RKO and LOVO expressing CHDH cells treated with 1,4‐DPCA or SP600125 or 1,4‐DPCA combine SP600125 were shown. Scale bar: 50 µm. (D) Statistic results on wound closure in RKO and LOVO expressing CHDH cells treated with 1,4‐DPCA or SP600125 or 1,4‐DPCA combine SP600125 were shown. **p < 0.01, ***p < 0.001. (E) Statistic results on migrated cells in RKO and LOVO expressing CHDH cells treated with 1,4‐DPCA or SP600125 or 1,4‐DPCA combine SP600125 were shown. ***p < 0.001. (F) Immunofluorescence assay to detect F‐actin rearrangement in RKO and LOVO expressing CHDH cells treated with 1,4‐DPCA or SP600125 or 1,4‐DPCA combine SP600125 via phalloidin staining. Scale bar: 20 µm.

We then evaluated the effects treatment with combination of the P4HA and c‐Jun inhibitors on CHDH‐mediated CRC cell migration. RKO‐ and LOVO‐CHDH cells were treated with 1,4‐DPCA combined with SP600125 resulting in the successful inhibition of P4HAs and collagen I expression and c‐Jun activation (Figure 7A). The wound healing (Figure 7B,C) and transwell (Figure 7D,E) assays showed that combined 1,4‐DPCA and SP600125 treatment was more effective in eliminating CHDH‐mediated CRC cell migration than either inhibitor alone. The immunofluorescence staining assay showed that combined 1,4‐DPCA and SP600125 treatment was more effective in abolishing CHDH‐mediated increased lamellipodia or cluster formation than either inhibitor alone as well (Figure 7F). Collectively, these findings suggested that 1,4‐DPCA combined with SP600125 can effectively eliminate CHDH‐mediated CRC cell migration in vitro.

### 2.8 Combined 1,4‐DPCA and SP600125 treatment diminished CHDH‐mediated tumor metastasis and progression in vivo

**FIGURE 8:**
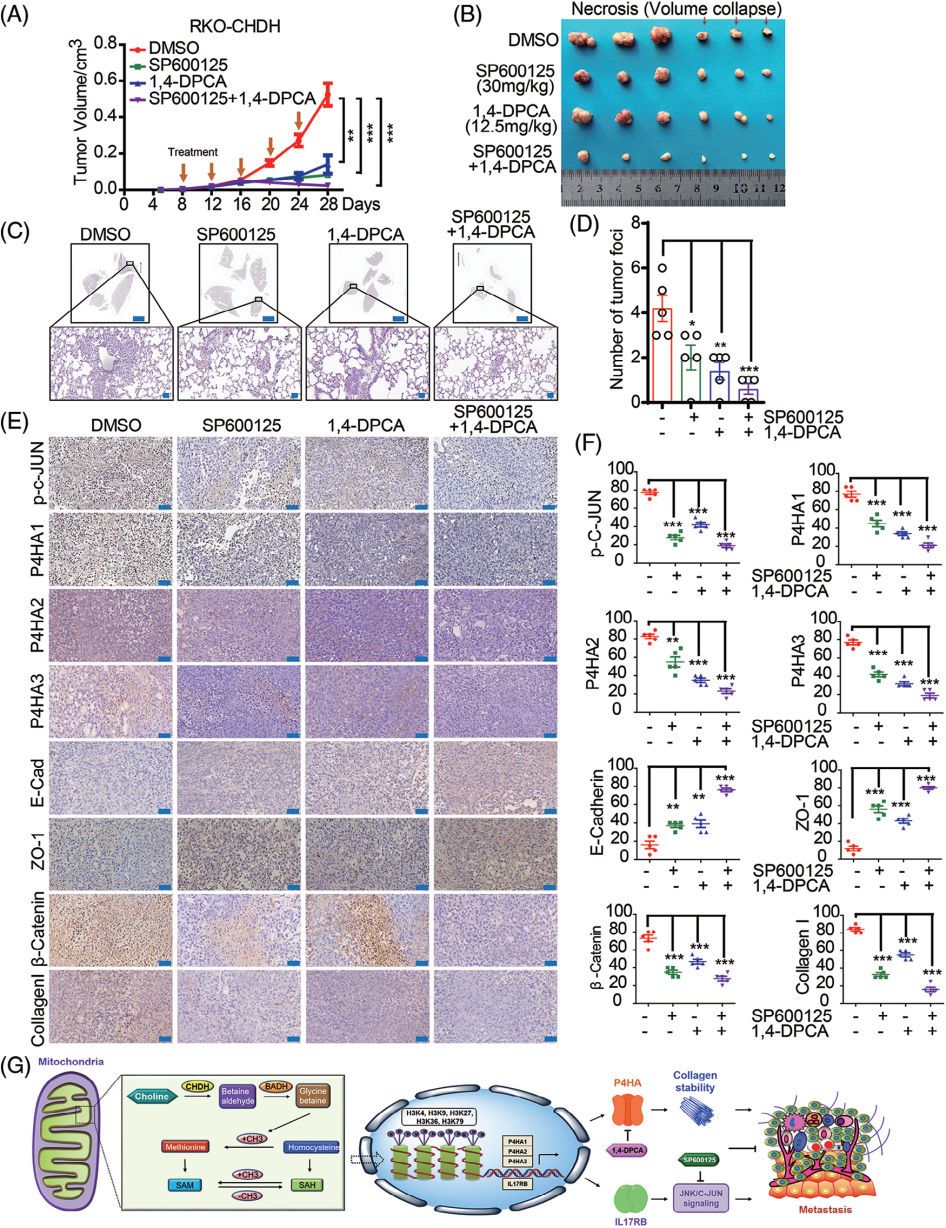
1,4‐Dihydrophenonthrolin‐4‐one‐3‐carboxylic acid (1,4‐DPCA) combined with SP600125 diminishes choline dehydrogenase (CHDH)‐mediated tumor metastasis and progression more effectively in vivo. (A) Stabilized RKO‐CHDH cells were injected subcutaneously at the fourth pair of fat pads in 5‐week‐old nude mice and treated with 1,4‐DPCA or SP600125 or 1,4‐DPCA in combination with SP600125 8 days after injection, the tumor growth curves were measured. **p < 0.01, ***p < 0.001. (B) The tumors separated from different group mice were shown. (C) Representative images of hematoxylin and eosin (H&E) staining on lungs from different groups were shown. (D) Statistic results of metastatic nodules in the lungs from various groups were shown. *p < 0.05, **p < 0.01, ***p < 0.001. (E) Immunohistochemistry (IHC) staining assay was conducted to check the expression of p‐c‐Jun, prolyl 4‐hydroxylase α‐subunit1/2/3 (P4HA1/2/3), ZO‐1, E‐cadherin, β‐catenin, and collagen I in various group mice tumors. Scale bar: 50 µm. (F) The statistical results of p‐c‐Jun, P4HA1/2/3, ZO‐1, E‐cadherin, β‐catenin, and collagen I positive cells in various group mice tumors were shown. **p < 0.01, ***p < 0.001. (G) Proposed model of CHDH in maintaining colorectal cancer (CRC) metastasis.

Since we demonstrated that combined inhibition with 1,4‐DPCA and SP600125 abrogated CHDH‐mediated CRC cell migration in vitro, we next validated these results in a xenograft mouse model in vivo. RKO‐CHDH stable cell lines were injected into the fourth fat pad of nude mice and treated 9 days after injection with 1,4‐DPCA, SP600125, or 1,4‐DPCA combined with SP600125. As shown in Figure 8A,B, 1,4‐DPCA combined with SP600125 was more effective at abolishing CHDH‐mediated tumor growth and tumor volume promotion than either inhibitor alone. H&E staining of mouse lungs displayed a decrease in metastatic nodules and areas in the 1,4‐DPCA, SP600125, and 1,4‐DPCA combined with SP600125 groups relative to the control (Figure 8C,D). The increased expression of epithelial markers, and reduced expression of mesenchymal markers were observed in the inhibitor treatment groups relative to that in the control group by IHC staining (Figure 8E,F). These findings reveal that 1,4‐DPCA combined with SP600125 diminishes CHDH‐mediated CRC tumor metastasis and progression in vivo.

## 3 DISCUSSION

CRC accounts for approximately 10% of all cancers and cancer‐related deaths annually worldwide.^34^ Metastasis, a hallmark of cancer, accounts for over 90% of cancer patient mortality. Metastases are systemic diseases, requiring a systemic treatment approach; screening, chemotherapy, targeted therapy, and immunotherapy is the mainstay for the prevention and treatment of metastatic CRC.^35^ Identifying new targets for early diagnosis, understanding their molecular mechanisms, and developing targeted therapy strategies are crucial for providing theoretical foundations and effective treatments for metastatic CRC. Therefore, we aimed to investigate the feasibility of the target molecule CHDH as a new marker for CRC metastasis. According to our results, CHDH was upregulated in colorectal cancer tissues compared to normal tissues and positively correlated with TNM stage. This is also consistent with results from online databases, which highlights the possibility that CHDH mediates CRC metastasis. However, the current studies lack a CHDH serum assay of patients with tumors. In future study, we will collect patient serum samples to determine whether there is secretory CHDH protein expression, which may be useful for early screening for CRC.

CHDH exerts a vital role in the pathogenesis and progression of several tumor types. For example, Ma et al.^36^ found that in breast cancer CHDH expression was regulated by estrogen and that its expression correlates with breast cancer prognosis. Zhang et al.^20^ demonstrated that low CHDH expression may be associated with poor overall survival in ccRCC patients by constructing a prognostic risk model for ccRCC associated with metabolic reprogramming. In studies on gastric cancer^37^ and hepatocellular carcinoma,^38^ high CHDH expression was found to indicate poor prognosis and shorter overall survival. However, the function of CHDH in CRC has not been reported, and its relationship with tumor metastasis is unclear. Here, we comprehensively explored the function of CHDH in CRC and found that CHDH not only promotes cell migration but also cell proliferation and cell cycle (data not shown). This result was also validated in mice in vivo, where tumor volume was remarkably increased in the CHDH expressed group. Since the function of CHDH in CRC has not been investigated, our study may broaden the understanding and perspective of CHDH function.

CHDH, a key enzyme associated with mitochondrial metabolism, localized to the inner mitochondrial membrane. The process by which CHDH influences molecular expression and activates downstream signaling pathways is intricate and complex. In this study, we first demonstrated that overexpressed CHDH is indeed localized in mitochondria, and we hypothesized that CHDH affects the translational equilibrium of the alpha coreceptor, leading to altered histone methylation levels, which in turn regulates downstream target genes such as *P4HA1‐3* and *IL17RB*. Of course this process was not rigorous and treatment with histone methylase inhibitors may be required to validate the results. To further investigate the mechanism of CHDH, in our future work we will present RNA‐sequencing analyses performed to identify relevant downstream target genes and signaling pathways.

Studies related to collagen P4HA in tumors have been commonplace. For example, P4HA1 stabilizes HIF‐1α expression and maintains chemotherapy tolerance in TNBC,^23^ P4HA2 and P4HA3 facilitate tumor malignancy progression. Specifically, P4HA2 regulates collagen deposition in breast cancer,^27^ and P4HA3 induces TGF‐β/Smad signaling activation to facilitate CRC cell growth and metastasis.^39^ However, the involvement of P4HA in CHDH‐driven CRC progression remains unreported. In our study, we found that P4HA1/2/3 were highly expressed in CRC tissues using IHC staining (data not shown). As a potentially relevant gene for CHDH, the expression of P4HA1/2/3 varied with CHDH expression. Our results suggest that CHDH expression affects the trimethylation status of histone H3, which in turn regulates the expression of P4HA. Regarding the role of P4HA in CHDH‐mediated CRC metastasis, we also demonstrated that P4HA expression may promote collagen I expression through hydroxylation, which in turn regulates the metastatic microenvironment and promotes metastasis. Additionally, application of the specific P4HA inhibitor 1,4‐DPCA revealed that P4HA plays an indispensable role in CRC metastasis. This study provides novel insights and evidence for the development of relevant therapies targeting CHDH.

C‐Jun has been widely studied in tumors as a downstream transcription factor in multiple signaling pathways.^30^ In our study, using protein kinase microarray analysis, we found for the first time that CHDH could activate c‐Jun and thus promote CRC metastasis. To further validate this result, we utilized small‐molecule inhibitors of c‐Jun activation and found that c‐Jun activation is essential for CHDH‐mediated CRC metastasis. Furthermore, we found that CHDH promotes IL17RB expression by affecting histone H3 trimethylation, which, in turn, activates c‐Jun. This could uncover a new mechanism through which CHDH facilitates tumor progression.

The ultimate goal for research on CHDH‐mediated CRC metastasis is to provide novel approaches for the treatment of clinical metastatic CRC. In this study, we also attempted targeted therapy against the CHDH‐mediated signaling pathway. We used the P4HA inhibitor 1,4‐DPCA and the C‐JUN inhibitor SP600125 to treat colorectal cancer metastasis, either separately or in combination, and the results showed favorable therapeutic effects on CHDH‐mediated metastasis. We expect that the combination of these two small‐molecule inhibitors can be used to eliminate or inhibit the progression of metastatic CRC when high CHDH expression is confirmed by serum or pathologic methods in the future clinical treatment.

In conclusion, we proposed a novel finding that CHDH, as one of the major enzymes of the methyl donor pathway, affects histone H3 trimethylation, which in turn regulates the expression of the downstream target genes P4HAs and IL17RB. The P4HAs stabilize and promotes collagen I expression, and IL17RB promotes downstream c‐Jun activation, which together contribute to the metastasis of CRC. We also demonstrated that 1,4‐DPCA targets P4HAs and SP600125 targets c‐Jun, both of which block CHDH‐mediated CRC metastasis (Figure 8G). Our findings indicate that CHDH could serve as a novel therapeutic target in the treatment of metastatic CRC.

## 4 MATERIALS AND METHODS

### 4.1 Online public database analyses

CHDH expression in normal and cancerous colon tissues was analyzed using the TIMER and UALCAN online public databases. GENEMANIA was used to detect gene sets related to CHDH function. TNMplot was used to analyze the expression of CHDH and IL17RB in CRC.

### 4.2 Human CRC clinical tissues

The human CRC clinical tissues we used in the study were obtained from patients undergoing surgery at Jining Medical University Affiliated Hospital between 2016 and 2019 (pathological details are listed in Tables S1 and S2). TNM staging of the obtained tissues was performed according to the American Joint Committee on Cancer guidelines. This study was approved by the Ethics Committee of Jining Medical University.^40^

### 4.3 Vector construction

A CHDH‐specific shRNA oligo was designed and synthesized (Table S3). The shRNA was ligated into the pLV‐H1‐EF1α‐Puro vector (Cat. #B19, Biosettia) to obtain the final pLV‐H1‐shCHDH‐EF1α‐Puro vector.^41^ The CHDH overexpression vector pLVML‐3×Flag‐CHDH‐Puro was obtained from Fenghui Biology and was generated using direct synthesis.

### 4.4 Immunohistochemistry

Human CRC tissue microarrays were purchased from Avilabio (Cat. #col11061, Cat. #col11034). Mouse tumor tissue sections were obtained by tissue dehydration, embedding, and sectioning. IHC analysis was conducted according to a previously described protocol.^41^, ^42^ The associated antibodies used were listed in Table S4.

### 4.5 Cell culture

Complete L15 medium was used to culture SW620 CRC cells. Complete McCoy's 5 medium (Gibco) was used to culture of HCT116 cells. Complete Dulbecco's modified Eagle medium (HyClone) was used for the culture of RKO and LOVO cells. All medium and supplements of the medium were purchased from Gibco. All cell lines were purchased from the American Type Culture Collection. Cell culture methods were according to previously described protocols.^40^, ^41^ The cells used were genetically profiled using the polymorphic short tandem repeat (STR) method at the Forensic Science Center of Jining Medical University and tested to ensure mycoplasma negativity.

### 4.6 Cell migration assay

The procedures of wound‐healing assay and transwell assay were followed according to previously described methods, materials.^43^

### 4.7 Western blotting

The western blotting procedure was followed according to a previously described method.^44^ All primary antibodies we used are listed in Table S4.

### 4.8 Human phosphokinase array

The Human Phosphokinase Array Kit was purchased from R&D Systems (Cat. #ARY003C), then was used to quantify relative kinase and protein phosphorylation levels following the manufacturer's instructions.^40^, ^41^

### 4.9 Cytoskeleton staining

Twenty thousand CRC cells were placed onto 24‐well plate special slides, fixed with 4% paraformaldehyde, washed with phosphate buffered saline (PBS), and stained with 5 µg/mL RED555‐phalloidin (Beyotime). Images were obtained with confocal microscope 80 × objective (Olympus).^45^

### 4.10 Immunoprecipitation

RKO‐CHDH and LOVO‐CHDH cell protein lysates were incubated with the corresponding antibodies at 4°C overnight, and then the mixtures were incubated with A/G agarose beads (Santa Cruz, sc‐2001) at 4°C for 3–5 h. The pellet was centrifuged at 3000 rpm for 60 s and washed carefully with precooled radio immunoprecipitation assay lysis buffer (RIPA) lysate. Western blot was performed by adding loading buffer and boiling.

### 4.11 In vivo experiments

Five‐week‐old female nude mice were randomly assigned to groups (*n* ≥ 5). Each mouse was injected subcutaneously with 3 × 10^6^ cells at the fourth pair of fat pads. The resulting tumor size was measured using calipers and volume calculated as follows: volume (mm^3^) = (width^2^ × length)/2. Tumor measurements were performed by analysts blinded to the treatment group. After a period ranging from 26 to 28 days, the mice were sacrificed, the tumor and lung tissues were isolated. Primary tumors and related lung tissues were formalin‐fixed, paraffin‐embedded, and sectioned for further analysis.

For inhibitor treatment, nude mice were injected with 3 × 10^6^ CRC cells. Eight days after injection, the mice were administered intraperitoneal injections of dimethyl sulfoxide, SP600125 (30 mg/kg every 3 days), or 1,4‐DPCA (12.5 mg/kg every 3 days) for a total of five injections.

For the tail vein‐mouse lung metastasis model, 5‐week‐old female nude mice were randomly assigned to groups (*n* ≥ 5). A total of 1 × 10^6^ cells/100 µL were injected via the tail vein. After 33 days, the mice were sacrificed and the lung tissue was isolated, formalin‐fixed, paraffin‐embedded, and sectioned for further analysis.

### 4.12 Statistical analysis

The GraphPad Prism9 software was used to perform data analysis. Values are expressed as mean ± standard error of the mean (SEM). Student's *t*‐test was used to calculate *p* values for two groups of data, and one‐way ANOVA was used for *p* values for more than two groups of data. *p* < 0.05 was set as statistically significant.^46^

## AUTHOR CONTRIBUTIONS

S.W.Z. designed the experiments, Y.X.W., L.Y.F., and S.X.Z. performed the experiments. W.S.Y., D.W.F., Y.C.L., and J.X.Y. prepared the materials and helped to conduct the experiments. Z.Z.Q. provided and helped to test the inhibitors. Z.X.Y. assisted in the analysis of the data. H.Y.M. helped to collect the patient's tumor tissue samples. Y.X.W. and S.W.Z. wrote the manuscript. H.Y.M., Z.X.Y., and S.W.Z. revised the manuscript, provided the funding for the experiments. All the authors have read and approved the final manuscript.

## CONFLICT OF INTEREST STATEMENT

The authors declare no conflicts of interest.

## ETHICS STATEMENT

Ethics in Tumor sample tissues: our institution has obtained informed consent from the patients, and the informed consent number is the patient ID number in Tables S1 and S2. This study was approved by the Institutional Ethics Committee of the Jining Medical University (Number: JNMC‐2023‐YX‐008).

Animal studies: The study was conducted according to established animal welfare guidelines and approved by Institutional Animal Committee of the Jining Medical University (Number: JNMC‐2023‐DW‐036).

## Supporting information



Supporting Information

## Data Availability

The publicly available data used in this study are available in the UALCAN (https://ualcan.path.uab.edu/) and TNMplot (https://tnmplot.com/analysis/), respectively. The data that support the findings of this study are available from the corresponding author upon reasonable request.
