# Supplementary material for: Prolyl 4‐hydroxylase α‐subunit family regulation of type I collagen deposition and IL17RB/c‐Jun activation synergistically mediate choline dehydrogenase promotion of colorectal cancer metastasis
Source: MedComm (2020). 2025 Jan 3;6(1):e70007. doi: 10.1002/mco2.70007 (PMC11702389; doi:10.1002/mco2.70007)
Supplement: Supplementary file 1 — Supporting Information [file MCO2-6-e70007-s001.pdf]

Supplementary Materials and Figures

Prolyl 4-hydroxylase α-subunit family regulation of type I collagen deposition and IL-17RB/c-Jun activation synergistically mediate choline dehydrogenase promotion of colorectal cancer metastasis

Xiaowen Yang[1, 2], Yifei Li[2], Xinzhuang Shen[2], Shuying Wang[3], Zhuqing Zhang[4], Wenfei Du[2], Chenglong Yang[2], Xinyu Jiang[2], Xiaoyuan Zhang[2], Yongming Huang[5, *], Wenzhi Shen[1, 2, *]

[1] Cheeloo College of Medicine, Shandong University, Jinan, 250012, China.

[2] Shandong Provincial Precision Medicine Laboratory for Chronic Non-communicable Diseases, Institute of Precision Medicine, Jining Medical University, Jining, 272067, China

[3] Department of Oncology and Southwest Cancer Center, Southwest Hospital, Third Military Medical University (Army Medical University), Chongqing, 400038, China

[4] Department of oncology, The Seventh Medical Center of Chinese PLA General Hospital

[5] Department of General Surgery, Affiliated Hospital of Jining Medical University, Jining Medical University, Jining, 272000, China

***Corresponding authors:**

Wenzhi Shen

Cheeloo College of Medicine, Shandong University, Jinan, 250012, China

Shandong Provincial Precision Medicine Laboratory for Chronic Non-communicable Diseases, Institute of Precision Medicine, Jining Medical University, Jining, 272067, China

E-mail: shenwenzhi@mail.jnmc.edu.cn

Yongming Huang

Department of General Surgery, Affiliated Hospital of Jining Medical University, Jining Medical University, Jining, 272000, China
E-mail: huangyongming@mail.jnmc.edu.cn

Running title: P4HA and c-Jun in CHDH-mediated CRC metastasis

Figure S1

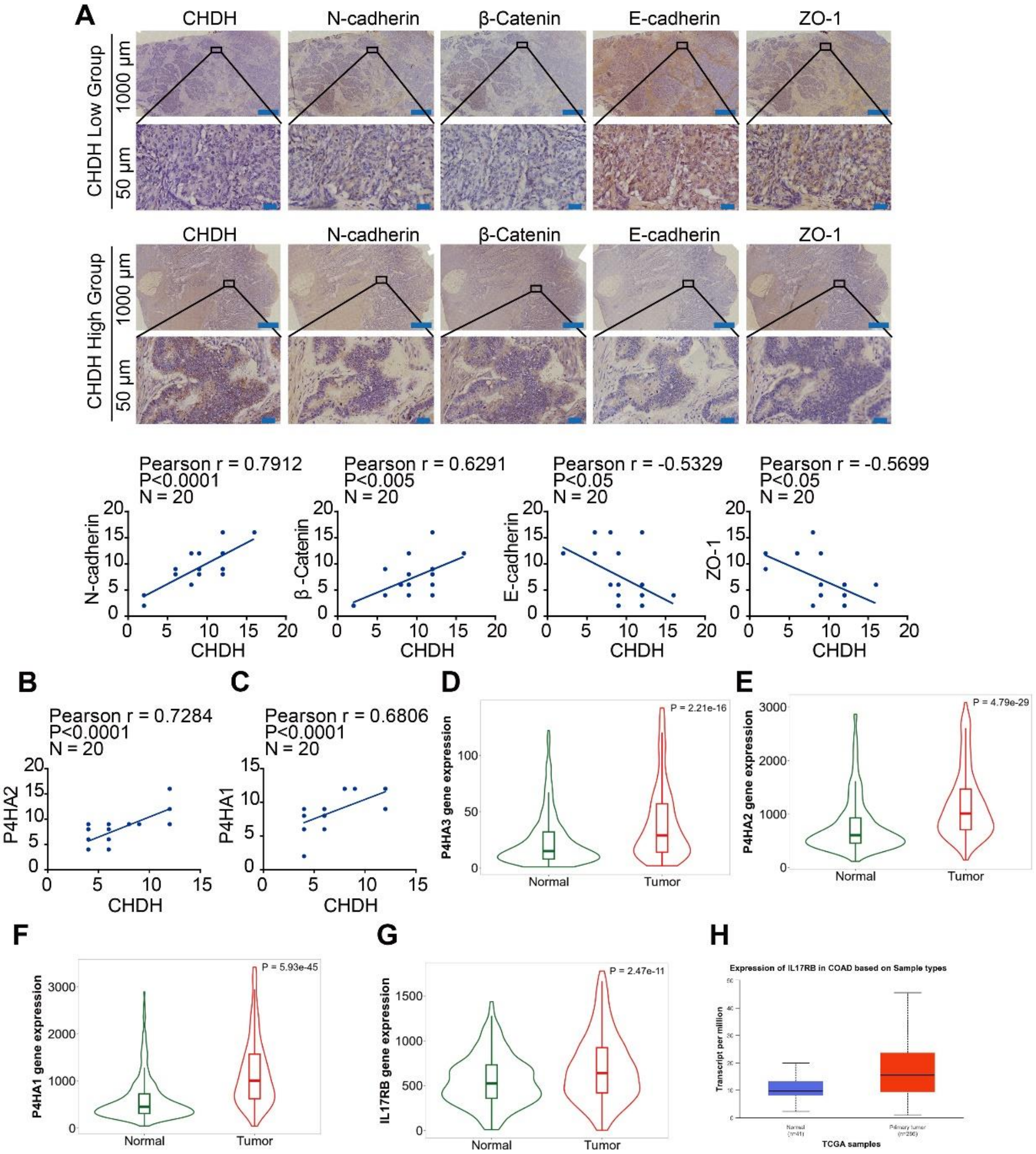

Figure S1. The correlation between CHDH and EMT markers and the expression of P4HA1/2/3 and IL17RB in CRC tissues. A. The correlation results between CHDH and EMT markers in human CRC tissues were shown. **B.** The correlation results between CHDH and P4HA2 in human CRC tissues were shown. **C.** The correlation results between CHDH and P4HA1 in human CRC tissues were shown. **D & E & F.** TNMplot online database was used to analyze the transcriptional expression of P4HA1, P4HA2 and P4HA3 in colon cancers (cancer versus normal tissue, $p < 0.05$). **G & H.**

TNMplot and UALCAN online databases were used to analyze the transcriptional expression of IL17RB in colon cancers (cancer versus normal tissue, $p < 0.05$).

Figure S2

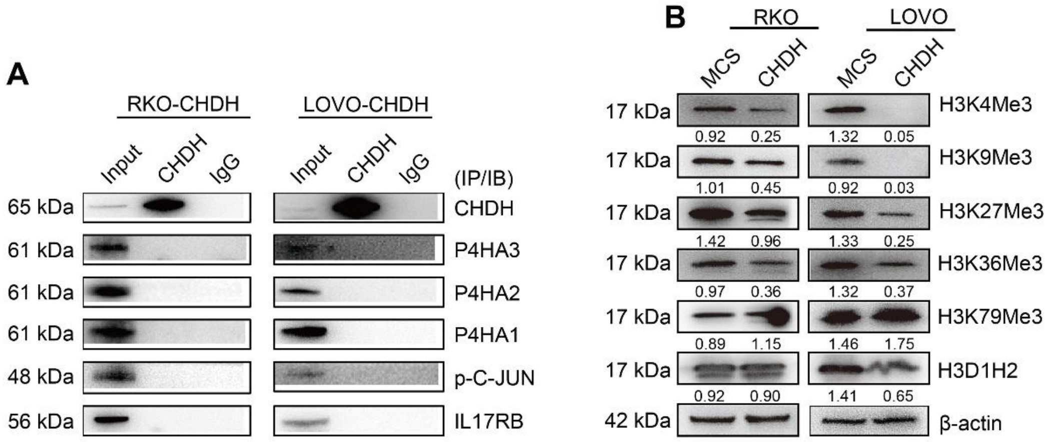

Figure S2. A. Interaction of CHDH with P4HAs, c-JUN and IL17RB was detected by immunoprecipitation**. B.** Western blot analysis the expression of histone H3 trimethylation in RKO and LOVO expressing CHDH or MCS cells.

Figure S3

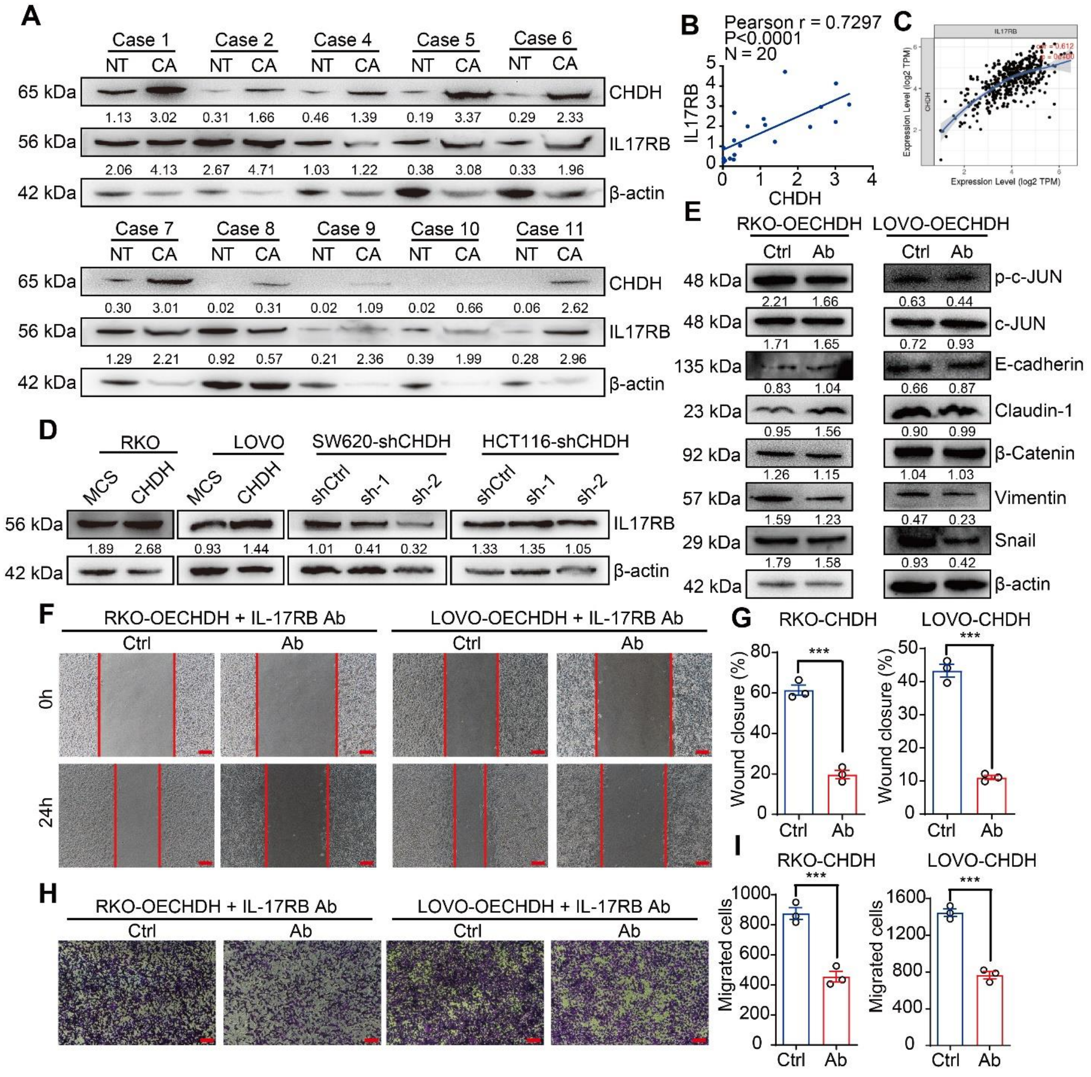

Figure S3. CHDH promotes IL17RB expression to mediate c-Jun activation and CRC cell migration *in vitro*

A. Western blot analysis of IL17RB expression in CHDH high-expressed colon cancer tissue and adjacent normal tissue, NT means normal tissue, CA means cancer tissue. **B.** The co-expression of CHDH and IL17RB in human CRC tissues were shown. **C.** TIMER online databases were used to analyze the co-expression of CHDH and IL17RB in human CRC tissues. **D.** Western blot analysis the expression of IL17RB in CHDH knockdown or overexpression cells. **E.** Western blot analysis the expression of c-Jun,

p-c-Jun and EMT markers in CHDH overexpression cells treated with IL17RB antibody. **F & G.** Representative images of wound healing assay and statistic results on wound closure in RKO and LOVO expressing CHDH cells treated with IgG (Ctrl) or IL17RB antibody were shown, Ab means IL17RB antibody. Scale bar: 50 μm, ***$p < 0.001$. **H & I.** Representative images of trans-well assay and statistic results on migrated cells in RKO and LOVO expressing CHDH treated with IgG (Ctrl) or IL17RB antibody were shown. Scale bar: 50 μm, ***$p < 0.001$.

Table S1. Patients information

Sample Number	ID Number	Sex	Age	Organ	Pathology Diagnosis	Type	Sampling Time	Blood
#1	3001518255	M	63	Colon	Adenocarcinoma	Malignant	16th Jul, 2018	No
#2	3001309502	M	62	Colon	Adenocarcinoma	Malignant	27th Aug, 2018	No
#3	3000517436	M	64	Colon	Adenocarcinoma	Malignant	31th May, 2017	No
#4	1000053719	F	67	Colon	Adenocarcinoma	Malignant	19th Mar, 2018	No
#5	3001338158	M	62	Colon	Adenocarcinoma	Malignant	2th Jul, 2018	No
#6	3001415877	M	64	Colon	Adenocarcinoma	Malignant	30th Aug, 2018	No
#7	3001609755	M	49	Colon	Adenocarcinoma	Malignant	20th Sep, 2018	No
#8	3001814784	M	64	Colon	Adenocarcinoma	Malignant	23th Jan, 2019	No
#9	3000272378	F	62	Colon	Adenocarcinoma	Malignant	8th Dec, 2018	No
#10	1004917055	M	74	Colon	Adenocarcinoma	Malignant	6th Sep, 2018	No
#11	3001592115	M	47	Colon	Adenocarcinoma	Malignant	18th Oct, 2018	No
#12	3001249891	M	75	Colon	Adenocarcinoma	Malignant	6th Sep, 2018	No

#13	3000756788	M	50	Colon	Adenocarcinoma	Malignant	4th Oct, 2017	No
#14	2414269925	M	42	Colon	Adenocarcinoma	Malignant	13th Oct, 2018	No
#15	3001340879	M	69	Colon	Adenocarcinoma	Malignant	27th Oct, 2018	No
#16	1005132835	F	67	Colon	Adenocarcinoma	Malignant	26th Apr, 2016	No

Table S2. Tissue information for 20 consecutively sectioned patients

Sample Number	ID Number	Sex	Age	Organ	Pathology Diagnosis	Type	Metastasis to lymph nodes
#20-1	3002001029	M	64	Colon	Adenocarcinoma	Malignant	Yes
#20-2	2889018401	M	67	Colon	Adenocarcinoma	Malignant	Yes
#20-3	10013401081	F	87	Colon	Adenocarcinoma	Malignant	Yes
#20-4	2889004377	F	77	Colon	Adenocarcinoma	Malignant	Yes
#20-5	3002045621	F	60	Colon	Adenocarcinoma	Malignant	Yes
#20-6	3002045519	F	62	Colon	Adenocarcinoma	Malignant	Yes
#20-7	3001992830	F	72	Colon	Adenocarcinoma	Malignant	Yes
#20-8	3002035205	F	62	Colon	Adenocarcinoma	Malignant	Yes
#20-9	3001687942	M	53	Colon	Adenocarcinoma	Malignant	Yes
#20-10	3002038194	F	83	Colon	Adenocarcinoma	Malignant	Yes
#20-11	2888996727	M	57	Colon	Adenocarcinoma	Malignant	Yes
#20-12	3002012709	F	67	Colon	Adenocarcinoma	Malignant	Yes

#20-13	3001917170	F	69	Colon	Adenocarcinoma	Malignant	Yes
#20-14	3002012689	M	60	Colon	Adenocarcinoma	Malignant	Yes
#20-15	2888997411	M	74	Colon	Adenocarcinoma	Malignant	Yes
#20-16	3001982603	F	36	Colon	Adenocarcinoma	Malignant	Yes
#20-17	2888985466	M	57	Colon	Adenocarcinoma	Malignant	Yes
#20-18	3001922516	M	56	Colon	Adenocarcinoma	Malignant	Yes
#20-19	3001995892	M	72	Colon	Adenocarcinoma	Malignant	Yes
#20-20	3001738993	M	49	Colon	Adenocarcinoma	Malignant	Yes

Table S3. Primer sequences

Name	Sequence
CHDH-shRNA-1	AAAAGGACATGACCATCCATGAAGGTTGGATCCAACCTTCATGGATGGTCATGTCC
CHDH-shRNA-2	AAAAGCTTGTGAGCAGGGTGCTATTTTGGATCCAAAATAGCACCCTGCTCACAAGC
shCtrl	AAAAGCTACACTATCGAGCAATTTTGGATCCAAAATTGCTCGATAGTGTAGC

Table S4. Antibodies List

Antibody		Clone, Cat #	Vendor	City, State, Country
Flag	Rabbit monoclonal	14793	Cell Signal Technology	Danvers, MA, USA
β-actin	Mouse monoclonal	sc-47778	Santa Cruz Biotechnology	Santa Cruz, CA, USA
CHDH	Mouse monoclonal	sc-393885	Santa Cruz Biotechnology	Santa Cruz, CA, USA
β-actin	Mouse monoclonal	sc-47778	Santa Cruz Biotechnology	Santa Cruz, CA, USA
Epithelial-Mesenchymal Transition (EMT) Antibody Sampler Kit	Rabbit monoclonal	9782	Cell Signal Technology	Danvers, MA, USA
COV Ⅳ	Rabbit monoclonal	A11631	ABclonal	Wuhan, China
P4HA1	Rabbit polyclonal	12658-1-AP	Proteintech	Wuhan, China
P4HA2	Rabbit polyclonal	13759-1-AP	Proteintech	Wuhan, China

P4HA3	Rabbit polyclonal	Ab101657	Abcam	Hong Kong, China
IL17RB	Rabbit polyclonal	20673-1-AP	Proteintech	Wuhan, China
P4HA3	Rabbit	PK25535	Abmart	Shanghai, China
Collagen Type I	Rabbit polyclonal	14695-1-AP	Proteintech	Wuhan, China
Tri-Methyl Histone H3Ab Sampler Kit	Rabbit monoclonal	9783	Cell Signal Technology	Danvers, MA, USA
P-c-Jun	Rabbit monoclonal	91952	Cell Signal Technology	Danvers, MA, USA
JUN	Mouse monoclonal	66313-1-Ig	Proteintech	Wuhan, China
IL-17RB	Mouse monoclonal	MAB1207	R&D	Shanghai, China
